# Cupping for patients with chronic urticaria: A systematic review protocol

**DOI:** 10.1097/MD.0000000000017115

**Published:** 2019-09-20

**Authors:** Xianjun Xiao, Yunzhou Shi, Leixiao Zhang, Wei Cao, Ying Liu, Siyuan Zhou, Mingling Chen, Qianhua Zheng, Ying Li

**Affiliations:** a Acupuncture and Tuina School, Chengdu University of Traditional Chinese Medicine; b Rehabilitation Department, The People's Hospital of Jianyang City; c Dermatological Department, Affiliated Hospital of Chengdu University of Traditional Chinese Medicine, Chengdu, Sichuan, China.

**Keywords:** chronic urticaria, cupping, protocol, systematic review

## Abstract

**Background:**

The program aims to evaluate the effectiveness and safety of cupping in patients with chronic urticaria (CU).

**Methods:**

We will search the databases including PubMed, Medline, Embase, the Cochrane Central Register of Controlled Trials (CENTRAL), Web of Science, China Science Journal Database, China National Knowledge Infrastructure, Wan-fang Database, and China Biomedical Literature Database from their inception to May 2019. In addition, we will manually search the list of medical journals as a supplement. The clinical randomized controlled trials or quasi-randomized controlled trials related to cupping for the treatment of CU will be included in the study. Data were synthesized by using a fixed-effect model or random effect model depend on the heterogeneity test. The primary outcome is the total effective rate. Secondary outcomes include skin disease quality of life index scores, adverse events, and recurrence rates. RevMan V.5.3 statistical software will be used for meta-analysis. If it is not appropriate for a meta-analysis, then a descriptive analysis will be conducted. Data synthesis will use the risk ratio and the standardized or weighted average difference of continuous data to represent the results.

**Results:**

This study will provide a comprehensive review of the available evidence to assess the effectiveness and safety of cupping for patients with CU.

**Conclusion:**

This systematic review (SR) will provide evidence to judge whether cupping is an effective intervention for patients with CU.

**Ethics and dissemination:**

The protocol of the SR does not require ethical approval because it does not involve humans. We will publish this article in peer-reviewed journals and presented at relevant conferences.

**Systematic review registration:**

PROSPERO, CRD42019137451

## 1 Introduction

Chronic urticaria (CU) is a condition characterized by the repeated occurrence of wheals (hives), angioedema, or both for >6 weeks.^[1]^ CU, including chronic spontaneous urticaria and chronic inducible urticaria, is one of the most frequent skin disorders which affects all age groups^[2,3]^ and has a female predominance.^[4–6]^ The prevalence of CU is estimated at approximately 1% of the world-wide population.^[7]^ For many patients with CU, The average duration of CU is reported lasting 2 to 5 years^[8–10]^ or longer.^[11]^ The effect of CU on patients can be significant. Some studies^[7,12,13]^ indicated that patients with CU had been suffering from anxiety, depression, irritability, or social dysfunction. Patients with CU frequently use several healthcare resources,^[14]^ which is costly for society.^[7]^

According to the treatment guidelines,^[1]^ the second-generation H1-antihistamines (sgAH) was the first-line recommended treatment. If CU symptoms were inadequate controlled, quadrupling the dosage of sgAH was recommended as second-line treatment, and omalizumab was recommended as third-line treatment. However, >60% of patients remained symptomatic despite treatment with sgAH at the licensed dose.^[15,16]^ Several reports indicated that no significant difference in efficacy between normal doses and high doses of sgAH.^[16–18]^ Omalizumab (anti-IgE) has been shown to be effective and safe in the treatment of CU.^[19–21]^ But the high cost of medical care imposes a heavy burden on patients and healthcare resources.^[22]^ As patients were still symptomatic despite treatment, many sought alternative medical therapies.^[7]^

Acupuncture is an important part of complementary and alternative medicine, which is widely used in the treatment of urticaria due to confirmed efficacy and few adverse effects.^[23]^ Cupping therapy (CT) is a kind of acupuncture which has a long history in Asia, especially in China.^[24,25]^ CT can be divided mainly into 2 styles: dry cupping and wet cupping.^[26]^ Dry cupping utilizes a glass or bamboo cup to create suction on the skin. Wet cupping involves creating tiny wounds on the skin before the cupping procedure, and the therapy is accompanied by the loss of blood from the wound.^[27,28]^ In recent years, CT has attracted much attention and has been widely used in dermatology.^[29]^ Some clinical reports suggest that CT is effective in treating CU in China.^[30–32]^ So far, there are no systematic reviews (SRs) of CT for CU. It is necessary for us to investigate the evidence of CT for CU. Therefore, we present the protocol of our proposed SR in CT for patients with CU.

## 2 Methods and analysis

### 2.1 Study registration

This SR was registered in International Prospective Register of Systematic Reviews (PROSPERO) as CRD42019137451. The protocol is structured complying with the Preferred Reporting Items for Systematic Reviews and Meta-analysis Protocols and Cochrane handbook.^[33]^ Before the start of the SR, consistency training will be conducted to ensure that all the reviewers have a basic understanding of the background, purpose, and process of the review.

### 2.2 Criteria for including studies

#### 2.2.1 Types of studies

Randomized controlled clinical trials (RCT) and quasirandomized controlled trials (q-RCT) will be included. Other types of literature will be excluded. Owing to the language restriction of our researchers, we will limit the language of search literature to Chinese and English.

#### 2.2.2 Types of participants

Eligible patients in our SR, regardless of race, sex, age, and education status, must comply with the European Academy of Allergology and Clinical Immunology, the Global Allergy and Asthma European Network, World Allergy Organization (EAACI/GA2LEN/EDF/WAO) guidelines^[1]^ or the Chinese Guidelines about the diagnosis and treatment of urticaria.^[34]^

#### 2.2.3 Types of interventions and comparisons

Eligible interventions in the experimental group include dry cupping and wet cupping. The cans include glass cans, bamboo cans, pottery cans, metal cans, plastic cans, etc. Intervention measures should be either cupping alone or combined with other methods to treat CU. If combined with other methods, only the control group with the same intervention measures as the experimental group will be included. If certain drug ingredients were added to the cans, they would be excluded. The following treatment comparisons will be investigated:

1. CT versus no treatment.
2. CT versus other active therapies.
3. CT plus active therapy versus the same active therapies.
4. CT versus placebo or sham CT.

### 2.3 Types of outcome measures

#### 2.3.1 The primary outcomes

The clinical total effective rate will be assessed through the primary outcome. According to the severity of clinical symptoms using 4 scores; the total score is the sum of the individual scores. Symptom Score Reducing Index = (total score before treatment-total score after treatment)/total score before treatment × 100%.

#### 2.3.2 Secondary outcomes

1. Skin disease quality of life index score.
2. Adverse events.
3. Recurrence rate during the follow-up period.

### 2.4 Search strategy

#### 2.4.1 Electronic searches

We will electronically search the following databases from their inception to May 1, 2019: PubMed, Medline, Embase, the Cochrane Central Register of Controlled Trials (CENTRAL), Web of Science, China Science Journal Database, China National Knowledge Infrastructure, Wan-fang Database, and China Biomedical Literature Database.

The following search terms will be used: Urticaria, Chronic urticaria, Nettle-rash, Hives, Rubella, Wind cluster, Angioedema, Cupping, Cupping therapy, Dry cupping, Wet cupping, Bloodletting, Pricking cupping, and pricking blood therapy. The example search strategy in Table 1 will be used for PubMed. This search strategy will be slightly modified and used in several other databases.

**Table 1:**
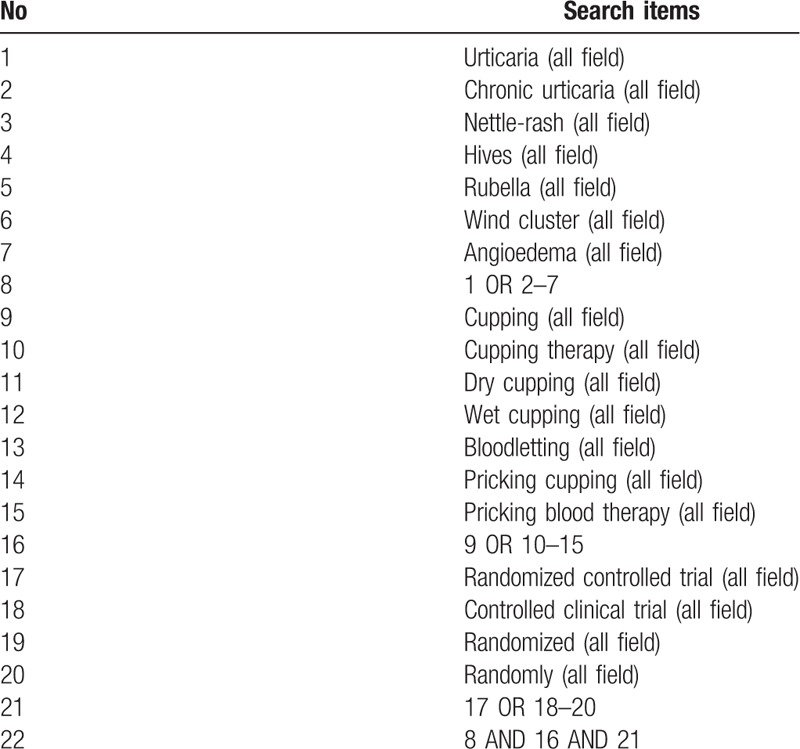
Search strategy used in PubMed.

#### 2.4.2 Searching other resources

We will review the reference lists of relevant RCTs and meta-analysis related to CT for potentially eligible studies. We will also search a reference list for identifying published journals, books, conference articles, and gray literature related to this research topic.

### 2.5 Data collection and analysis

#### 2.5.1 Selection of studies

**Figure 1:**
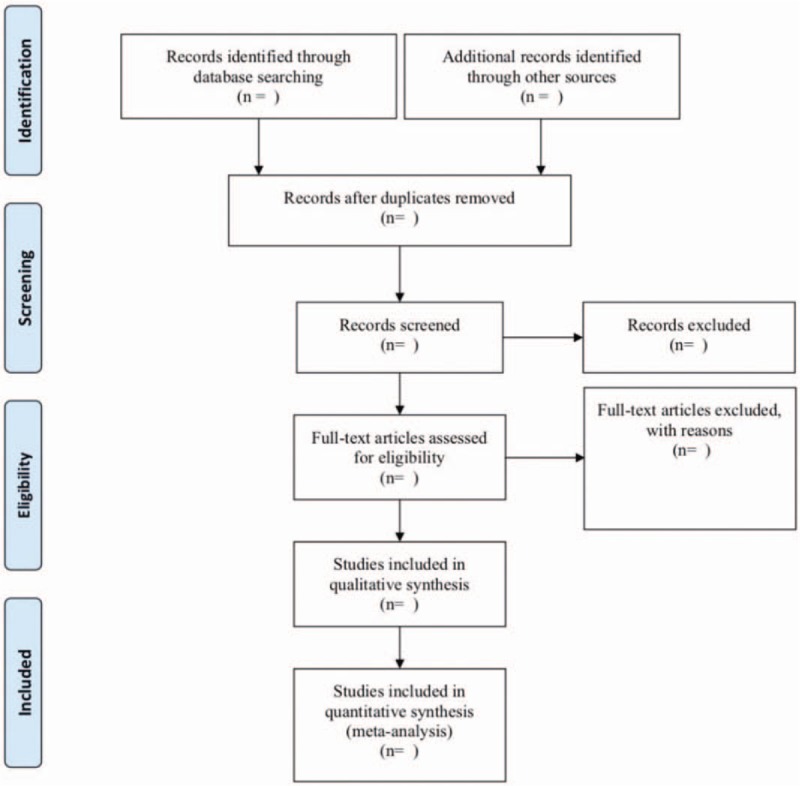
The PRISMA flow diagram of the study selection process. PRISMA = preferred reporting items for systematic reviews and meta-analysis protocols.

The bibliographies yielded by the literature search will be imported into Noteexpress software Version 2.6.1 (Aegean Sea software company Beijing, China) for management. Two review authors (YS and LZ) will independently review and screen the titles, abstracts, and keywords of all retrieved studies to confirm eligible trials. Reviewers will obtain full-text reports for further assessment. The explanations for exclusion will be recorded in an excel data set. Inter-reviewer (YS and LZ) disagreements will be resolved by discussion or by a third party's arbitration (XX). The research flow chart is shown in Figure 1.

#### 2.5.2 Data extraction and management

Two reviewers (WC and YL) will independently check the eligibility of the included studies and extract data according to a predesigned data extraction form. After a common consensus is reached, they will make an Excel to extract the following information: general information, participant, methods, interventions, outcome, results, adverse events, main conclusions, conflicts of interest, ethical approval, and other information. In the process, any discrepancy will be resolved through discussion between the 2 authors, or judged by the third author (YL). If the reported data are not sufficient, we will contact the author of the experiment for consultation and solution.

#### 2.5.3 Assessment of risk of bias in included studies

Two authors (SZ and QZ) will independently use the Risk of bias tool in Cochrane Manual V.5.1.0 to evaluate the bias risk of each included studies. The following domains will be accessed: random sequence generation, allocation sequence concealment, blinding of participants and personnel and outcome assessors, incomplete outcome data, selective outcome reporting, and other sources of bias. The assessment results will be divided into 3 levels: low risk, high risk, and uncertain risk. In the process, the discrepancy will be discussed to reach an agreement. If necessary, the discrepancy will be judged by the third author (YL).

#### 2.5.4 Measures of treatment effect

For continuous data, mean difference (MD) and 95% confidence intervals (CIs) will be used to measure the treatment effect. For dichotomous data, risk ratio (RR) with 95% CIs will be used to measure the treatment effect.

#### 2.5.5 Dealing with missing data

We will try to contact the first or corresponding authors of the included studies to get missing or insufficient trial data by email or telephone. If additional data are not available, we will analyze the existing data, and discuss the potential impact of missing data.

#### 2.5.6 Assessment of heterogeneity

The heterogeneity of data will be tested by calculating the value of the *I*^2^ statistic. When the *I*^2^ value is <50%, the study is not considered to have large heterogeneous. When the *I*^2^ value exceeds 50%, there is significant statistical heterogeneity among the trials, and meta-analysis will not be performed. At that time, sensitive analysis or subgroup analysis will be conducted.

#### 2.5.7 Assessment of reporting biases

If the sufficient number of included studies (at least 10 trials) are available, we will use Funnel charts to assess reporting biases. An Egger test will be conducted to access the funnel plot asymmetry.^[35]^

#### 2.5.8 Date synthesis

RevMan V.5.3 statistical software will be applied for data synthesis. The results will be expressed as RR and the standardized or weighted average difference of continuous data. The specific methods are as follows: If the *I*^2^ test is <50%, the fixed-effects model will be used for data synthesis. If the *I*^2^ test is from 50% to 75%, the random-effects model will be conducted for data synthesis. If the *I*^2^ test is >75%, we will investigate possible reasons from both clinical and methodological perspectives to conduct subgroup analysis. If data cannot be synthesized, we will provide a descriptive analysis to solve this problem.

#### 2.5.9 Subgroup analysis

A subgroup analysis will be conducted to explore the potential causes of heterogeneity if necessary. The following subgroup analysis plan will be considered.

(1) Different types of cupping (dry cupping or wet cupping).
(2) Different types of intervention forms (cupping alone or combined with other active treatments).

#### 2.5.10 Sensitivity analysis

In order to identify the robustness of the primary results, we will conduct a sensitivity analysis. Methodological quality, sample size, and missing data which will be the principal decision nodes. The meta-analysis will be reused, and more inferior quality studies will be excluded. The results will be compared and discussed according to the results.

#### 2.5.11 Grading the quality of evidence

The quality of SRs will be evaluated by using the Grading of Recommendations Assessment, Development, and Evaluation. Five downgrading factors including risk of bias, inconsistency, indirectness, imprecision, and publication bias will be assessed. The assessment results will be divided into 4 levels: high, moderate, low, or very low.

## 3 Discussion

CU is a prevalent and relevant dermatological problem that affects human health. Many patients and some dermatologists are unsatisfactory for the therapeutic pathways. CT is an important therapy in the world medical practice. Currently, there are some clinical trials to confirm the efficacy and safety of CT for CU. However, there is not an SR related to CT for CU has been published in the world. Whether it can be a new choice in clinical treatment, this SR will summarize current evidence on the effectiveness and safety of CT for CU. We hope this review will help clinicians make better decisions.

## Author contributions

**Conceptualization:** Xianjun Xiao.

**Data curation:** Yunzhou Shi, Leixiao Zhang, Wei Cao, Ying Liu, Qianhua Zheng.

**Formal analysis:** Xianjun Xiao, Siyuan Zhou.

**Investigation:** Wei Cao.

**Methodology:** Xianjun Xiao, Yunzhou Shi, Ying Liu.

**Project administration:** Xianjun Xiao, Yunzhou Shi.

**Supervision:** Ying Li.

**Writing – original draft:** Xianjun Xiao.

**Writing – review & editing:** Mingling Chen.
